# 

**Published:** 1908-07-04

**Authors:** 


CONTENTS.

| | PAGE |
|---|---|
| **The Temperamental Factor in Disease** ... ... | 353 |
| **Suicide** ... ... ... ... ... ... ... ... ... ... ... | 354 |
| **Annotations**— | |
| The Age of Forty—The Birthday Honours—Art and Prudery... | 355 |
| **Medical Opinion and Movement** ... ... ... ... ... | 356 |
| **Hospital Clinics**— | |
| Chronic Laryngitis. By Harold Barwell, M.B., F.R.C.S. ... | 359 |
| **Special Article**— | |
| The Nervous Manifestations of Rickets ... ... ... ... | 362 |
| **Medicine**— | |
| The Sequelæ of Enteric Fever—V. ... ... ... ... ... | 363 |
| X-rays and Hepatic Abscesses... ... ... ... ... ... | 364 |
| **Points in Surgery**— | |
| Acute Retention of Urine—I. ... ... ... ... ... ... | 365 |
| Some Aspects of Intestinal Obstruction ... ... ... ... | 366 |
| **Diseases of Children**— | |
| The Early Signs of Measles ... ... ... ... ... ... | 367 |
| **The General Practitioner's Column**— | |
| The Local Treatment of Pain in Gout ... ... ... ... | 368 |
| **Therapeutics**— | |
| Prescriptions and Dispensing ... ... ... ... ... ... | 369 |
| Secret Remedies and Branded Products—VII. ... ... ... | 370 |
| **Pathology**— | |
| Tubercle Bacilli in Cardiac Vegetations ... ... ... ... | 371 |
| **Cases for Diagnosis** ... ... ... ... ... ... ... ... | 372 |
| **Hospital Administration**— | |
| The Civic Hospital of Venice ... ... ... ... ... ... | 373 |
| New Appliances and Things Medical ... ... ... ... | 374 |
| **Editor's Letter-Box** ... ... ... ... ... ... ... | 374 |
| **News and Coming Events** ... ... ... ... ... | 375 |
| **Nursing Administration**— | |
| The Duty of Hospitals in regard to Thrift ... ... ... | 377 |
| **League of Mercy Garden Party** ... ... ... ... | 378 |
| **The Graduates' Medical Schools** ... ... ... ... | 378 |

